# The Relationships between Various Factors and Sleep Status: A Cross-Sectional Study among Healthy Saudi Adults

**DOI:** 10.3390/nu15184090

**Published:** 2023-09-21

**Authors:** Sara AL-Musharaf, Basmah Albedair, Waad Alfawaz, Madhawi Aldhwayan, Ghadeer S. Aljuraiban

**Affiliations:** 1 Department of Community Health Sciences, College of Applied Medical Sciences, King Saud University, Riyadh 11451, Saudi Arabia; 443203479@student.ksu.edu.sa (B.A.); walfawaz@ksu.edu.sa (W.A.); maldhwayan@ksu.edu.sa (M.A.); galjuraiban@ksu.edu.sa (G.S.A.); 2 Center of Excellence in Biotechnology Research, King Saud University, Riyadh 11451, Saudi Arabia

**Keywords:** Pittsburgh Sleep Quality Index, sleep quality, sleep duration, sleep status, neck circumference

## Abstract

Impaired sleep can adversely affect daily life. This study assesses the association between different factors and sleep status among apparently healthy Saudi adults. In total, 478 adults were included in this study. Data on anthropometrics, body composition, stress scales, physical activity, and dietary habits were collected. Fasting blood glucose and lipid profile were measured. Sleep quality and duration were assessed using the Pittsburgh Sleep Quality Index. Larger neck circumference (NC) was associated with short sleep duration (odds ratio (OR) 1.23; 95% confidence interval (CI) [1.08, 1.41]; *p* = 0.002). Higher triglyceride levels were associated with poor sleep quality (OR 1.01; 95% CI [1.002, 1.02]; *p* = 0.019) and short sleep duration (OR 1.01; 95% CI [1.004, 1.02]; *p* = 0.005). Stress was a risk factor for poor sleep quality (OR 1.15; 95% CI [1.09, 1.22]; *p* < 0.001). Being married was significantly associated with good sleep quality (OR 2.97; 95% CI [1.32, 6.71]; *p* = 0.009), while being single was correlated with longer sleep duration (OR 0.46; 95% CI [0.22, 0.96]; *p* = 0.039). Other factors such as having a larger waist circumference and more muscle mass were protective factors against poor sleep quality and/or short sleep duration. In conclusion, a larger NC is suggested as a risk factor for short sleep duration and a higher triglyceride level for both short and poor sleep among healthy Saudis. Investigating the factors associated with sleep status may help alleviate sleep disturbances and improve overall health. Further studies are needed to confirm causality using objective sleep measures.

## 1. Introduction

Sleep is a physiological necessity for humans and is essential for maintaining health and wellbeing [1]. During the COVID-19 outbreak, sleep disturbances affected 27.6% of the global population [2]. Previous studies have indicated a high prevalence of sleep disturbances in different countries, including 35.4% among the Chinese adult population [3], 77.8% among Canadians [4] and 55.5% among the Saudi population [5]. A study conducted in Saudi Arabia reported that 49.6% of adults had a short sleep duration (≈<7 h/day) [6]. The recommended amount of sleep duration for Saudi adults is about 7–9 h of quality sleep per night [7].

Short and disturbed sleep may increase the risk of obesity [8], cardiovascular disease (CVD) [9], cancer [10], and depression [11]. Sleeping less than 6 h per night has been shown to decrease cognitive performance and disrupt mood [12], increase the risk of dementia [13], and contribute to higher mortality rates [14].

Several studies have demonstrated that factors such as age [15], anthropometric indices, and adiposity [16,17] can affect sleep status. For example, a larger neck circumference (NC) was found to be inversely associated with sleep duration [18], sleep quality [19], and excessive daytime sleepiness (EDS) [20]. In addition, waist circumference (WC) and body fat percentage were positively correlated with sleep quality score [18,21].

Another factor that may negatively influence sleep is stress, as it plays a significant role in reducing sleep quality [22,23]. It has been reported that higher stress scores are associated with poor sleep, with a sensitivity of 64% and a specificity of 72% among the adult population [24].

Moreover, physical activity has a bidirectional relationship with sleep [25]. Studies have suggested that physical activity could improve sleep quality in several ways, such as by regulating cortisol, which is a key biomarker of sleep [26,27].

Furthermore, recent studies on the relationship between specific serum biomarkers, such as levels of triglyceride (TG) [28] and deficiencies of certain serum vitamins and minerals [29,30], and sleep are gaining attention. In this context, a cohort study observed that higher TG levels were significantly associated with poor sleep quality among adults [31].

Investigating the factors associated with sleep status will help improve sleep status and, consequently, enhance the overall quality of life. Several studies have investigated the association between different factors and sleep [21,23,27] among specific groups, including patients, students, and employees. However, few studies have assessed the relationship between various factors and sleep status in the healthy Saudi adult population, and the measuring of NC related to sleep has exclusively been carried out among sleep apnea patients in Saudi Arabia. We hypothesized that several lifestyle factors have a relationship with sleep status. Hence, the aim of this study was to examine the association between different factors (including sociodemographic factors, dietary data, physical activity, stress, and serum biomarkers of fasting glucose and lipid profiles) and sleep status among apparently healthy Saudi adults.

## 2. Materials and Methods

### 2.1. Study Design

A cross-sectional study was conducted among Saudi adults between December 2021 and March 2022. In total, 534 participants were recruited from different shopping malls in Riyadh, Saudi Arabia, though 47 were excluded due either to incomplete data or because they were pregnant or lactating women, using vitamins or antihypertensive medications, or had a history of dyslipidemia, CVD, stroke, or diabetes. The remaining total was 487 males and females aged ≥ 18 years, who were included in the study. 

This study was approved by the Institutional Ethics Committee of King Khalid University Hospital (KSU-IRB No. E-21-5852). All participants provided informed consent before participating and were informed that they had the right to withdraw from the study at any time.

### 2.2. Sample Size

The sample size was calculated using the G*Power software version 3.1.9.7. The minimum required sample size was 415 participants, considering an alpha error of 5%, a power of 80%, and a minimal model R-squared of 5% and allowing 20 predictors to be included in the model. A total of 487 participants met the eligibility criteria and were included in this study.

### 2.3. Data Collection

The recruitment was from different shopping malls in Riyadh via the simple random sampling method. We obtained permission to collect the data at each site. The study had a portable booth at each location; each booth included three main areas. The first area explains the study objectives and obtains consent from interested participants. Then, there are two separate interview areas for data collection (semi-isolated to assure the participants’ privacy). The interview areas contain a sphygmomanometer, non-stretchable measuring tape, and the instrument for bioelectrical impedance analysis in a 25 m^2^ space (5 × 5).

Trained staff collected sociodemographic data and anthropometric measurements. Other data, such as sleep status [32], stress [33], physical activity [34] and dietary data [35], were collected using standardized and validated Arabic questionnaires. Subsequently, each participant was scheduled for an appointment at the study lab to undergo a blood test.

#### 2.3.1. Anthropometric Measurements

Anthropometric data were assessed using standardized procedures in accordance with the study protocol. All participants were informed they needed to be on an empty stomach and go to the toilet before having the measurements. All measurements were taken twice, and then the average was used for the final analysis. The participants’ body weights (kg), body fat percentages, and muscle mass were measured using the bioelectrical impedance analysis (BIA) (770 Bioelectrical Impedance Analyzer, InBody, Seoul, Republic of Korea). Height (cm) was measured using a digital electronic device. Body mass index (BMI) was calculated by dividing the weight in kilograms by the square of the height in meters (kg/m^2^), using the Centers for Disease Control and Prevention (CDC) BMI classification [36]. WC, NC, and hip circumference (in cm) were measured using non-stretchable plastic tape. In addition, the waist-to-hip ratio was calculated by dividing the mean WC by the average hip circumference [37].

#### 2.3.2. Biochemical Blood Analysis

During the lab visit, blood samples were collected by a trained nurse after a fasting period of 8–10 h. The levels of fasting glucose and lipid profile (including high-density lipoprotein (HDL), total cholesterol (TC), and TG levels) were measured using the VITROS ECi Immunodiagnostic System and Roche COBAS e411. Low-density lipoprotein (LDL) cholesterol was calculated using the Friedewald formula (LDL = TC-(HDL + 0.20 × TG)) [38]. The Roche COBAS e411 Analyzer was used to measure insulin levels.

#### 2.3.3. Sociodemographic Characteristics

The questionnaire to collect sociodemographic characteristics was adapted from a survey previously published in Saudi Arabia [39]. It contains sociodemographic and socioeconomic data, including the participants’ city of residence, academic level, income, occupation, marital status, medical history, and smoking status.

#### 2.3.4. Sleep Index

The Pittsburgh Sleep Quality Index (PSQI) (validated Arabic version) was used to evaluate the participants’ sleep status [32]. The PSQI consists of 19 individual items or questions that are categorized into seven elements. The following are the elements: subjective sleep quality, sleep latency, sleep duration, sleep efficiency, sleep disturbances, use of sleep medication, and daytime dysfunction [32].

Each element has four choices with scores ranging from 0 to 3 (0 = not during the past month, 1 = less than once a week, 2 = once or twice a week, and 3 = three or more times a week). The component scores are then summed to obtain an overall score, which ranges from 0 to 21. The higher scores indicate more significant sleep problems, specifically, a total score higher than five indicates poor sleep quality [32]. Sleep duration was classified into four categories (>7 h, 6–7 h, 5–6 h, and <5 h) based on the PSQI classification [40].

#### 2.3.5. Stress Scale

The Perceived Stress Scale (PSS) (Arabic version) was used to assess the participants’ perceptions of stress over the past month. It comprises 10 questions with 5 response options each; these questions include several queries about participants’ experiences with stress. Higher scores represent a higher level of stress [33].

#### 2.3.6. Global Physical Activity Questionnaire

The Arabic-validated version of the Global Physical Activity Questionnaire (GPAQ) was used to assess physical activity [34]. The GPAQ covers four domains: occupational physical activity, transport-related physical activity, physical activity during discretionary or leisure time, and sedentary behavior. It includes the following elements of physical activity: intensity, duration, and frequency [34].

#### 2.3.7. Dietary Intake Assessment

To assess the participants’ dietary intake, we used the Saudi Food Frequency Questionnaire (FFQ) and conducted a 24 h dietary recall for 2 consecutive days [35]. On the recruitment day, a qualified clinical dietitian conducted face-to-face interviews to collect data using the Saudi FFQ, which consists of a list of approximately 133 traditional food items in Saudi Arabia. In addition, dietary recall of the past 24 h was obtained during the interview, and then a second recall was collected via phone [35]. The FFQ was analyzed using ESHA Food Processor SQL software version 16.0 (ESHA Research, Salem, UT, USA) based on frequency and portion size in grams (g). An analysis of macronutrients (carbohydrates, proteins, and fats) was also included in this study.

### 2.4. Statistical Analysis

Statistics version 25 was used for all statistical analyses.

The general characteristics of the participants are presented as mean ± standard deviation or percentage in Supplementary Table S1. The participants were stratified into two groups based on their sleep quality score: good or poor, as presented in Table 1. A PSQI score of less than five indicates good sleep quality, while a score equal to or greater than five indicates poor sleep quality.

The chi-squared test was used to compare categorical variables. Comparisons of sleep duration categories with other categorical variables such as sex, education, and family income were performed using the chi-squared test (crosstabulation), while the *t*-test was used to compare continuous data. Analysis of variance (ANOVA) was used to compare three or more means. Logistic regression was conducted to assess factors associated with the dichotomous PSQI (good vs. poor) as the outcome variable.

A multinomial logistic regression was performed to evaluate the factors associated with different sleep duration categories, with the category >7 h of sleep as the reference group. The variables adjusted in the regression analysis of sleep quality included age; marital status; income; stress; energy, carbohydrate, and protein intake; and BMI, WC, NC, TG, and HDL levels. In sleep duration models, we adjusted for sex; age; marital status; physical activity; stress; muscle mass; fat, carbohydrate, and protein intake; and BMI, WC, NC, fasting glucose, cholesterol, TG, and LDL levels. The regression analysis was conducted using three models (Model 1: 6–7 vs. >7 h of sleep; Model 2: 5–6 vs. >7 h of sleep; Model 3: <5 vs. >7 h of sleep). Factors that showed a *p* < 0.25 in the bivariate analysis were considered as independent variables in the final models. A *p* value less than 0.05 was deemed statistically significant.

## 3. Results

### 3.1. General Characteristics of the Participants

A total of 487 participants enrolled in this study, and 65.9% of them were females (Supplementary Table S1). The mean age was 35.19 ± 12.74 years. The majority (73.9%) had a university level of education. The average BMI was 27.77 ± 6.15 kg/m^2^ and the NC was 35.15 ± 4.07 cm. The mean score on the PSQI was 8.16 ± 3.44, which indicated poor sleep quality. Also, short sleep duration (less than 5 h) was most widely represented (31.1%) among the participants compared to other sleep duration categories. The majority of the participants were non-smokers. Additional details related to the characteristics of the participants are summarized in Supplementary Table S1.

### 3.2. Factors Associated with the Sleep Quality Scores

There was a significant difference between the mean scores of higher energy intake (*p* = 0.02), BMI (*p* < 0.001), waist circumference (*p* < 0.001), fat percentage (*p* < 0.001), TG (*p* < 0.001), and stress (*p* < 0.001) in those who had poor sleep quality compared to those who had good sleep quality (Table 1).

### 3.3. Multinomial Analysis of Factors Associated with the Dichotomous PSQI

Table 2 presents the odds of sleep quality (poor vs. good) and specific correlates. Higher carbohydrate intake (adjusted odd ratio (aOR) = 0.996, 95% confidence interval (CI) = 0.993; 0.999) was significantly associated with lower odds of having poor quality of sleep, whereas higher stress (aOR = 1.15, 95% CI = 1.09; 1.22), being single compared to being married (aOR = 2.97, 95% CI = 1.32; 6.71), and higher TG levels (aOR = 1.01, 95% CI = 1.002; 1.02) were significantly associated with higher odds of having poor quality of sleep (Table 2).

### 3.4. Factors Associated with Sleep Duration Categories

The bivariate analysis showed that carbohydrate and protein intake, muscle mass, and older age were higher in those who sleep 6–7 h per night. A higher BMI, waist circumference, and total MET score were significantly more likely in participants who sleep 5–6 h per night compared to the other groups. Finally, NC and waist-to-hip ratio were higher in those who sleep less than 5 h per night (Table 3).

### 3.5. Multinomial Logistic Regression of Factors Associated with Sleep Duration Categories

None of the variables were significantly associated with higher odds of sleeping 6–7 h per night (Table 4, Model 1). Being single vs. being married (aOR = 0.46) and having a higher average muscle mass (aOR = 0.96) were significantly associated with lower odds of sleeping 5–6 h compared to more than 7 h, whereas higher TG (aOR = 1.01) was significantly associated with higher odds of sleeping 5–6 h compared to more than 7 h (Table 4, Model 2). The third model compared participants who sleep less than 5 h per night to those who slept more than 7 h. The results showed that higher NC (aOR = 1.23) and higher TG levels (aOR = 1.01) were significantly associated with higher odds of sleeping less than 5 h, whereas a greater waist circumference (aOR = 0.93) and higher carbohydrate intake (aOR = 0.995) were significantly associated with lower odds of sleeping less than 5 h (Table 4, Model 3).

## 4. Discussion

In the current study, we investigated the association of sleep status with anthropometric, biochemical, and lifestyle factors. Marital status and stress were identified as the most significant factors affecting sleep quality. Moreover, a larger NC was found to be the most significant risk factor for short sleep duration (<5 h). Additionally, high TG levels were associated with poor sleep quality and short sleep duration. Other factors such as being single, having a larger WC, having more muscle mass, and having a higher carbohydrate intake were found to have a positive impact on sleep duration. Furthermore, our findings showed that individuals with a high PSQI score (8.16 ± 3.44) had poor sleep quality.

Interestingly, TG levels in our study were significantly associated with both sleep quality and duration. We observed that every 1-unit increase in TG levels increased the odds of poor sleep quality and short sleep duration by 1%. It is noteworthy that the average levels of TG for the sample were within the normal range. Several studies have reported associations between serum lipid profiles and sleep status among different populations [31,41,42,43]. However, the results of these studies have been inconsistent. In line with our results, a cohort study observed that poor sleep quality was significantly associated with higher TG levels among adults [31]. Moreover, high TG and low HDL-C levels were found to be significantly associated with short sleep duration among different population groups in the U.S. [42].

The pathophysiological mechanism that may explain the effect of serum lipids on sleep duration remains unclear. Chen et al. [44] proposed that an elevated TG level strongly influences sleep patterns through nocturia (excessive urination at night) [45], which, in turn, may disturb sleep [46]. On the other hand, it has been hypothesized that alterations in lipid levels could serve as early markers of impaired metabolism induced by poor sleep quality. In this regard, a community-based study among 400 middle-aged and elderly individuals evaluated the relationships between sleep duration and serum lipid profiles [41]. The study reported no significant association between TG level and sleep duration. The inconsistency among the studies may be attributed to differences in sample size, age groups, health status, and confounding variables. Therefore, further longitudinal studies are warranted to investigate this relationship.

Our study revealed that NC was significantly associated with sleep duration. Specifically, for every 1 cm increase in NC, there was a 23% higher likelihood of having a shorter sleep duration (<5 h). Limited studies have investigated the relationship between NC and sleep status [18,19,21,47], and some have indicated a significant association between larger NC and either poor sleep quality [18,19,21] or shorter sleep duration [18,47]. However, some of the participants were obese, and the majority were at a high risk of obstructive sleep apnea [19,21]. In agreement with our result, a cross-sectional study among 88 young, healthy adults concluded that larger NC readings had a significant negative correlation with total sleep duration [18]. This result remains significant when measuring sleep objectively; for example, Moraes et al. [47] proposed that short sleep duration was associated with a larger NC when the former was measured using actigraphy and polysomnography. Our study was adjusted for BMI, suggesting that an enlarged neck size causes narrowing of the upper airway, which could result in snoring or apnea. These symptoms, in turn, may disturb sleep and reduce its duration. A large NC is a recognized risk factor for obstructive sleep apnea [48,49]. Similar to our results, other studies did not find an association between NC and sleep quality [50,51] due to small sample sizes and other confounding factors [50].

We found a significant positive association between stress and poor sleep quality after accounting for confounding factors. For every 1-unit increase in stress score, the chances of poor sleep increased by 15%. Similar to our study, a cross-sectional study concluded that mental workload-related stress was associated with poorer sleep efficiency among healthy Japanese adults, as determined by actigraphy (aOR = 1.53; *p* < 0.01) [52]. Furthermore, it has been reported that healthy middle-aged adults experiencing psychological stress have lower sleep efficiency, shorter sleep duration, and longer sleep onset than non-stressed individuals [22]. This could be explained by the fact that elevated stress hormones, mainly cortisol, further disrupt sleep.

Regarding marital status, we found that single individuals had approximately a three-fold higher risk of poor sleep quality than married individuals. The same finding was reported in a community-based cross-sectional study conducted in China among adults aged 18 to 90 [53]. The study found that poorer sleep quality was associated with being single (aOR = 1.31, *p* = 0.034) [53]. This may be attributed to the fact that living with a partner is associated with greater social and emotional support than living alone [54]. Another study reported a positive correlation between loneliness and increased sleep disturbance [54]. However, our results showed that single participants had significantly longer sleep hours, with the finding that single participants were protected by 54% from shorter sleep duration compared to married individuals. Hence, singles have longer sleep hours but poorer sleep quality.

Our study reported that for every 1 cm increase in WC, the likelihood of sleeping more than 7 h increased by 7%. Similarly, a community-based study among 7094 Chinese adults showed that greater WC (≥80 cm) was associated with longer sleep duration among women but not men (OR = 1.30) [55]. Apparently, individuals who sleep for longer hours tend to lead a sedentary lifestyle and consume more snacks and may overestimate their actual sleep duration because they spend more time in bed [56,57,58]. The mechanism behind this could be due to pro-inflammatory cytokines associated with abdominal obesity, such as tumor necrosis factor-alpha and interleukin-6 (IL-6) [59,60]. These cytokines have been found to promote sleepiness, fatigue, and other health effects [61,62]. Moreover, higher IL-6 levels were found to be associated with poorer sleep quality among healthy adults [63]. In contrast, other studies showed either no association [64] or negative associations [18,65] between WC and sleep duration.

Furthermore, participants with higher muscle mass exhibited a 4% lower probability of experiencing shorter sleep duration (*p* = 0.014). More muscle mass is indicative of better physical fitness [66], which, in turn, is associated with longer sleep duration [67]. As mentioned previously, abdominal obesity was negatively associated with sleep duration [65], which is in line with our findings. Thus, higher muscle mass and lower body fat may contribute to increased sleeping hours.

With regard to dietary habits, our results suggest that for every 1 g increase in carbohydrate intake, the odds of sleeping more than 7 h and achieving good sleep quality increase by 0.5%. However, we did not find any significant relationship between protein and fat intake and sleep status. Evidence suggests that high carbohydrate intake is associated with reduced sleep-onset latency and slow-wave sleep as well as increased rapid eye movement (improved sleep architecture and quality) [68]. Additionally, a cross-sectional study conducted among 304 females reported that a low-carbohydrate diet was associated with improved sleep quality, which may be attributed to the reduction in inflammatory marker levels [69]. It is important to note that the study participants were women who were overweight or obese. Low-carb diets help increase protein intake, which is a rich source of tryptophan. Tryptophan can improve sleep quality by promoting the production of the sleep hormone melatonin. Melatonin is produced from serotonin, which is derived from tryptophan [69]. Carbohydrate intake increases brain tryptophan as a net result of the action of insulin. Insulin stimulates the uptake of large neutral amino acids (LNAAs) into skeletal muscle. LNAAs compete with tryptophan for transport to the brain. Tryptophan, on the other hand, is a precursor of serotonin, which affects sleep; therefore, the consumption of carbohydrates may improve sleep health [1].

### Strengths and Limitations

To our knowledge, this study is among the first few studies examining the associations between various factors and sleep status among apparently healthy Saudi adults. We used validated Arabic questionnaires and conducted face-to-face interviews to ensure higher accuracy. All anthropometric measurements were taken twice, and the dietary data were assessed using multiple methods. Additionally, we adjusted for confounding variables. However, this study also has some limitations that highlight the need for further research. First, the cross-sectional design could not confirm a causal relationship. Second, there was a possibility of recall bias, and third, the study did not investigate participants’ intake of coffee, tea, or energy drinks, which could have been associated with their sleep status. Finally, the study assessed sleep duration indirectly by using the components related to sleep duration in the PSQI, similar to previous studies [18,70]. However, measuring sleep objectively will give better results.

## 5. Conclusions

Our study showed that several risk factors are associated with poor sleep status. Specifically, being single and having stress were found to be the most significant risk factors for poor sleep quality, and a larger NC was found to be the most significant risk factor for shorter sleep duration. Moreover, higher TG levels were associated with both poor sleep quality and shorter sleep duration. Avoiding a sedentary lifestyle and engaging in regular physical activity, which includes building muscle mass and reducing body fat, have a positive impact on sleep status and overall quality of life. However, prospective studies with objective sleep measurements are needed to confirm a causal relationship.

## Figures and Tables

**Table 1:** Baseline characteristics of adults stratified by sleep quality *.

	PSQI Scores		
	Good Sleep (<5)	Poor Sleep (≥5)	*p*-Value
Age (years)	36.91 ± 12.59	34.87 ± 12.76	0.19
Sex			0.25
Males	47 (14.6%)	274 (85.4%)	
Females	31 (18.7%)	135 (81.3%)	
Education			0.34
Secondary or less	17 (13.4%)	110 (86.6%)	
University	61 (16.9%)	299 (83.1%)	
Family income (>10,000 SAR/month)			0.07
<10,000 (SAR)	22 (12.0%)	161 (88.0%)	
≥10,000 (SAR)	55 (18.2%)	247 (81.8%)	
Marital status			0.11
Married	44 (18.7%)	191 (81.3%)	
Single	34 (13.5%)	218 (86.5%)	
Stress (PSS)	12.49 ± 6.60	17.85 ± 6.98	<0.001
Anthropometrics			
BMI (kg/m^2^)	25.74 ± 5.30	28.15 ± 6.23	<0.001
NC (cm)	34.56 ± 3.87	35.26 ± 4.10	0.16
WC (cm)	82.55 ± 15.17	87.88 ± 16.78	<0.001
WHR (ratio)	0.84 ± 0.11	0.86 ± 0.11	0.05
Fat percentage %	32.63 ± 9.85	36.65 ± 10.60	<0.001
Muscle mass (kg)	34.15 ± 11.96	34.73 ± 11.35	0.68
Biochemical data			
Fasting glucose levels (mg/dL)	93.90 ± 18.94	92.63 ± 18.65	0.61
Fasting insulin levels (μU/mL)	9.63 ± 6.21	11.85 ± 19.24	0.36
Cholesterol (mg/dL)	181.68 ± 39.75	183.64 ± 40.94	0.72
TG (mg/dL)	73.73 ± 34.44	89.98 ± 59.35	<0.001
LDL (mg/dL)	107.61 ± 33.90	108.57 ± 33.30	0.83
HDL (mg/dL)	55.15 ± 15.83	52.07 ± 11.11	0.06
Diet			
Energy intake (kcal/day)	3824 ± 1630.38	4306 ± 1913.70	0.02
Fat (gm)	72 ± 38.17	70 ± 42.05	0.71
Carbohydrates (gm)	258 ± 138.40	233 ± 134.79	0.14
Proteins (gm)	79 ± 37.67	73 ± 43.44	0.21
Physical activity			
Total MET	1524.42 ± 1512.67	1513.27 ± 2824.86	0.97
Sitting	36.71 ± 13.46	38.05 ± 12.62	0.40
Smoking			
No	70 (16.6%)	352 (83.4%)	0.38
Yes	8 (12.3%)	57 (87.7%)	

* Data presented as mean ± standard deviation or (%); BMI: body mass index; HDL: high-density lipoprotein; LDL: low-density lipoprotein; MET: metabolic equivalent; NC: neck circumference; PSQI: Pittsburgh Sleep Quality Index; PSS: perceived stress scale; TG: triglycerides; WC: waist circumference; WHR: Waist-to-hip ratio.

**Table 2:** Multinomial logistic regression taking the dichotomous PSQI (poor vs. good) as the dependent variable using the ENTER method.

	aOR	95% CI	*p*-Value
Energy intake Kcal/day	1.00	1.00; 1.00	0.16
BMI (kg/m^2^)	1.09	0.99; 1.20	0.08
WC (cm)	1.05	1.00; 1.10	0.06
NC (cm)	0.90	0.79; 1.02	0.09
Carbohydrates (gm)	0.996	0.993; 0.999	<0.001
Proteins (gm)	1.00	0.99; 1.01	0.66
Stress	1.15	1.09; 1.22	<0.001
Age, years	0.98	0.95; 1.01	0.26
Income (≥10,000 vs. <10,000 *)	0.56	0.27; 1.17	0.12
Marital status (single vs. married *)	2.97	1.32; 6.71	<0.001
TG (mg/dL)	1.01	1.002; 1.02	0.01
HDL (mg/dL)	0.98	0.95; 1.01	0.14

aOR: adjusted odds ratio for all listed variables; CI: confidence interval; * reference number; BMI: body mass index; NC: neck circumference; WC: waist circumference; TG: triglycerides; HDL: high-density lipoprotein; PSQI: Pittsburgh Sleep Quality Index.

**Table 3:** Bivariate analysis of factors associated with sleep duration categories.

	Sleep Duration Categories				
	>7 h	6–7 h	5–6 h	<5 h	*p*-Value
Age (years)	32.46 ± 13.12	38.30 ± 12.87	36.20 ± 12.64	35.24 ± 12.09	<0.001
Sex					0.17
Males	97 (30.2%)	48 (15.0%)	76 (23.7%)	100 (31.2%)	
Females	36 (21.8%)	28 (17.0%)	50 (30.3%)	51 (30.9%)	
Education					0.54
Secondary or less	40 (31.5%)	19 (15.0%)	28 (22.0%)	40 (31.5%)	
University	93 (25.9%)	57 (15.9%)	98 (27.3%)	111 (30.9%)	
Family income (SAR/month)					0.34
<10,000 (SAR)	54 (29.7%)	25 (13.7%)	41 (22.5%)	62 (34.1%)	
≥10,000 (SAR)	78 (25.8%)	51 (16.9%)	84 (27.8%)	89 (29.5%)	
Marital status					<0.001
Married	48 (20.4%)	43 (18.3%)	69 (29.4%)	75 (31.9%)	
Single	85 (33.9%)	33 (13.1%)	57 (22.7%)	76 (30.3%)	
Anthropometrics					
BMI (kg/m^2^)	26.81 ± 5.88	26.83 ± 5.71	28.88 ± 6.11	28.18 ± 6.49	0.02
WC (cm)	84.09 ± 15.20	86.05 ± 16.27	90.16 ± 17.46	87.55 ± 17.03	0.02
NC (cm)	33.95 ± 3.54	34.77 ± 4.06	35.82 ± 4.33	35.83 ± 4.08	<0.0001
WHR (ratio)	0.83 ± 0.10	0.84 ± 0.11	0.87 ± 0.11	0.88 ± 0.12	<0.0001
Fat percentage %	35.94 ± 11.17	33.99 ± 10.01	36.76 ± 10.58	36.57 ± 10.26	0.28
Muscle mass (Kg)	34.96 ± 12.21	38.38 ± 13.25	32.92 ± 11.37	33.90 ± 9.35	<0.001
Diet					
Energy intake (kcal/day)	4095 ± 1773	4314 ± 2019.90	4442 ± 1993.69	4106 ± 1779.95	0.37
Fat (gm)	73 ± 38.60	80± 41.85	67 ± 35.65	65 ± 47.01	0.05
Carbohydrates (gm)	251 ± 121.65	262 ± 15	241 ± 126.77	206 ± 134.51	<0.001
Proteins (gm)	74 ± 41.41	86 ± 42.94	73 ± 40.85	67 ± 43.75	0.01
Labs					
Fasting glucose levels (mg/dL)	90.40 ± 15.33	91.05 ± 8.59	94.17 ± 19.69	94.98 ± 23.94	0.22
Fasting insulin levels (mg/dL)	10.73 ± 8.34	10.14 ± 9.50	14.34 ± 32.66	10.60 ± 7.07	0.35
Cholesterol (mg/dL)	177.09 ± 34.86	190.51 ± 45.82	186.40 ± 41.79	182.40 ± 41.41	0.16
TG (mg/dL)	71.20 ± 33.38	82.20 ± 33.80	99.70 ± 60.41	94.56 ± 73.58	<0.001
LDL (mg/dL)	102.17 ± 28.43	113.42 ± 36.22	111.35 ± 33.00	108.83 ± 35.75	0.11
HDL (mg/dL)	53.79 ± 14.88	53.46 ± 10.71	51.38 ± 10.38	51.97 ± 11.31	0.45
Physical Activity					
Total MET	1266.49 ± 1620.09	1741.89 ± 4150.20	2034.53 ± 3177.40	1175.71 ± 1765.39	0.03
Sitting	38.98 ± 12.86	36.48 ± 12.65	37.30 ± 12.27	38.04 ± 13.10	0.54
Stress (PSS)	17.27 ± 7.93	15.62 ± 8.07	16.50 ± 6.46	17.87 ± 6.54	0.12
Smoking					0.91
No	115 (27.3%)	64 (15.2%)	110 (26.1%)	132 (31.4%)	
Yes	18 (27.7%)	12 (18.5%)	16 (24.6%)	19 (29.2%)	

Data are presented as mean ± standard deviation or (%) and analyzed by ANOVA and chi-squared test. BMI: body mass index; NC: neck circumference; WC: waist circumference; WHR: waist-to-hip ratio; TG: triglycerides; HDL: high-density lipoprotein; LDL: low-density lipoprotein; MET: metabolic equivalent; PSS: perceived stress scale.

**Table 4:** Multinomial regression taking the low/high sleep quality as the dependent variable.

	Model 1			Model 2			Model 3		
Variables	aOR	95%CI	*p*	aOR	95%CI	*p*	aOR	95%CI	*p*
Gender	0.81	0.24; 2.74	0.73	0.39	0.13; 1.19	0.09	0.51	0.17; 1.56	0.23
Age	1.03	0.99; 1.06	0.10	0.99	0.96; 1.02	0.53	0.99	0.96; 1.02	0.40
BMI (kg/m^2^)	0.99	0.88; 1.12	0.91	1.07	0.96; 1.20	0.20	1.11	1.00; 1.24	0.05
WC (cm)	0.98	0.93; 1.03	0.37	0.96	0.91; 1.01	0.10	0.93	0.88; 0.97	<0.001
NC (cm)	1.01	0.86; 1.19	0.88	1.14	0.99; 1.31	0.07	1.23	1.08; 1.41	<0.001
Fat (gm)	1.00	0.99; 1.01	0.65	1.00	0.99; 1.01	0.90	1.00	0.99; 1.01	0.66
Carbohydrates (gm)	1.00	0.99; 1.004	0.74	1.00	0.99; 1.003	0.93	0.99	0.992; 0.999	0.01
Proteins (gm)	1.00	0.99; 1.01	0.36	0.99	0.98; 1.00	0.21	1.00	0.99; 1.01	0.82
Fasting glucose levels (mg/dL)	0.99	0.97; 1.02	0.47	0.99	0.97; 1.02	0.55	1.00	0.98; 1.02	0.91
Total MET	1.00	1.00; 1.00	0.28	1.00	1.00; 1.00	0.29	1.00	1.00; 1.00	0.14
Stress	0.99	0.95; 1.04	0.80	1.02	0.97; 1.07	0.41	1.04	0.99; 1.09	0.07
Marital status (single vs. married *)	0.65	0.29; 1.46	0.29	0.46	0.22; 0.96	0.03	0.70	0.34; 1.44	0.33
Average muscle mass	1.03	0.99; 1.06	0.12	0.96	0.93; 0.99	0.01	0.98	0.95; 1.01	0.20
Cholesterol (mg/dL)	1.01	0.99; 1.02	0.58	1.00	0.98; 1.02	0.93	0.99	0.98; 1.01	0.37
TG (mg/dL)	1.00	0.99; 1.02	0.52	1.01	1.00; 1.02	<0.001	1.01	1.004; 1.02	<0.001
LDL (mg/dL)	1.00	0.99; 1.02	0.67	1.00	0.99; 1.02	0.72	1.01	0.99; 1.03	0.26

aOR: adjusted odds ratio for all listed variables; CI: confidence interval; * reference group; BMI: body mass index; NC: neck circumference; WC: waist circumference; WHR: waist-to-hip ratio; TG: triglycerides; LDL: low-density lipoprotein; MET: metabolic equivalent. Model 1: 6–7 h of sleep vs. >7 h; Model 2: 5–6 h of sleep vs. >7 h; Model 3: <5 h of sleep vs. >7 h.

## Data Availability

Not applicable.

## Supplementary Materials

The following supporting information can be downloaded at: https://www.mdpi.com/article/10.3390/nu15184090/s1, Table S1: General characteristics of the participants (*n* = 487).
