# Supplementary material for: The Relationships between Various Factors and Sleep Status: A Cross-Sectional Study among Healthy Saudi Adults
Source: Nutrients. 2023 Sep 21;15(18):4090. doi: 10.3390/nu15184090 (PMC10536185; doi:10.3390/nu15184090)
Supplement: Supplementary file 1 [file nutrients-15-04090-s001.zip › nutrients-2586818-supplementary.pdf]

Table S1. General characteristics of the participants (n= 487).

Characteristics	n (%)
**Age** (years)	35.19 ± 12.74
**Sex**	
Males	166 (34.1%)
Females	321 (65.9%)
**Education**	
Secondary or less	127 (26.1%)
University	360 (73.9%)
**Family income (>10,000 SAR/month)**	
<10000 (SAR)	183 (37.7%)
≥10000 (SAR)	302 (62.3%)
**Marital status**	
Married	235 (48.3%)
Single	252 (51.7%)
**Anthropometrics**	**Mean ± SD**
BMI (kg/m^2)	27.77 ± 6.15
NC (cm)	35.15 ± 4.07
WC (cm)	87.03 ± 16.64
WHR (ratio)	0.86 ± 0.11
Fat percentage %	36.02 ± 10.58
Muscle mass (kg)	34.64 ± 11.44
**Labs**	
Fasting glucose levels (mg/dL)	92.85 ± 18.68
Fasting insulin levels (μU/mL)	11.47 ± 17.74
Cholesterol (mg/dL)	183.31 ± 40.70
Triglycerides (mg/dL)	87.24 ± 56.23
LDL (mg/dL)	108.41 ± 33.36
HDL (mg/dL)	52.59 ± 12.06
**Diet**	
Energy intake (kcal/day)	4229.06 ± 1878.03
Fat (gm)	70.13 ± 41.42
Carbohydrates (gm)	236.99 ± 135.53
Proteins (gm)	73.67 ± 42.59
**Physical activity**	
Total MET	1515.04 ± 2658.88
Sitting	37.84 ± 12.75
**Sleep**	
PSQI	8.16 ± 3.44
Sleep duration	
>7 hours	133 (27.4%)
6-7 hours	76 (15.6%)
5-6 hours	126 (25.9%)
<5 hours	151 (31.1%)
**Stress (PSS)**	16.99 ± 7.19
**Smoking**	
No	422 (86.7%)
Yes	65 (13.3%)

BMI: body mass index; NC: neck circumference; WC: waist circumference; WHR: Waist to hip ratio; HDL: high-density lipoprotein; LDL: low-density lipoprotein; MET: metabolic equivalent; PSQI: Pittsburgh Sleep Quality Index; PSS: perceived stress scale.
